# What can glove impression evidence reveal about assailants? A pilot study

**DOI:** 10.1080/20961790.2019.1684642

**Published:** 2019-11-29

**Authors:** Melad G. Paulis

**Affiliations:** a Department of Forensic Medicine and Clinical Toxicology, Faculty of Medicine, Minia University, Minia, Egypt; b Department of Internal Medicine, Faculty of Medicine, Mu’tah University, Mu’tah, Jordan

**Keywords:** Forensic sciences, glove, impressions, sex examination, stature examination

## Abstract

Impressions and marks are expected components of any crime scene. There is nothing more disappointing for fingerprint experts than finding glove marks at a crime scene. The forensic expert’s primary task in such cases is to compare the characteristic features of glove impressions with the characteristics of a suspect. The aim of the present study was to determine whether additional information could be obtained from glove prints. Specifically, whether they could be used to help to predict the sex and stature of a suspect was investigated, as was the potential for ascertaining additional information in cases where such prints were recovered from diverse objects with different diameters. Male and female participants wore latex gloves, and after ink was applied to the gloves they grasped objects of different diameters (2, 4, 6 and 8 cm). Impressions of gloved flat hands were also obtained. Phalangeal and finger lengths were measured digitally via software. Sex and stature were successfully estimated based on impressions derived from gloved flat hands and from prints on various grasped objects. A regression equation was developed for stature prediction, and a discriminant equation was developed for sex prediction.

## Introduction

Impressions and marks are expected components of any crime scene. Numerous things can produce impressions. The most common and extensively studied types of impressions are those made by footwear. Many studies have investigated personal identification and sex and stature estimation via footwear impressions [1–3]. Other sources of impressions are tool marks, tyre marks, and fabric impressions. Glove impressions or prints are a type of fabric print [4].

People wear gloves for protection from extreme weather, diseases, contamination, and potentially hazardous chemicals. There is now a general awareness of handprints among many lawbreakers, and accordingly many try to avoid depositing them at crime scenes. Because of this, finding a complete handprint at the scene of a crime perpetrated by such an offender is unusual [5]. Notably however, Fisher and Fisher [6] reported that offenders tend to believe that gloves offer complete security, and thus they utilise gloved hands without restraint. Accordingly, complete glove impressions can sometimes be found in obvious and easily accessible places at crime scenes.

Gloved hands carry dirt, dust and other materials from elsewhere. They can also contain residues from typical crime scene components such as blood or other body fluids. In accordance with Locard’s Exchange Principle [7], gloved hands leave impressions or marks when they touch other surfaces. These are positive impressions. Negative impressions can also occur when a gloved hand removes dust, blood or other materials from a surface [4].

Like fingerprints, glove impressions can be two-dimensional or three-dimensional depending on the nature of the surface they are left on. They can consist of patent marks and/or latent marks. Visible glove prints can be photographed and lifted using a black gelatin lifter. Latent impressions can be visualized with fingerprint powders [8].

Lambourne [9] described four types of glove marks that could be detected at crime scenes: leather, fine fabrics (such as cotton), coarse fabrics (such as wool), and rubber or latex. Latex is a natural rubber and a highly regular cis-1,4-polyisoprene produced by more than 400 different species of plants [10]. Criminals prefer latex gloves because they fit the hands tightly, facilitating a better grasp of objects [11, 12].

Glove marks obtained from a crime scene can provide information pertaining to the manufacturing features of the gloves worn during the crime. In addition, numerous features individualize each glove and make comparisons between prints and actual gloves procured as potential evidence possible. Such features include the material the glove was made from and how it was constructed, (*i.e.* by machine or handmade, stitched, knitted, moulded, or embossed). Acquired features such as holes, tears, or other imperfections also characterize each glove. Notably however, whether glove impressions can still have a role in crime scene investigations when there is no actual glove to use in comparisons remains to be thoroughly investigated.

Several reports indicate the usefulness of handprints for estimating stature and sex [13, 14]. In the real world, forensic experts are confronted with two challenges. The first is that most criminals use gloves to protect themselves from being identified via fingerprints left at the crime scene [11]. The second is related to the unfixed glove impression shape due to the high mobility of the hands [15]. Glove prints may be found on flat surfaces or on curved objects of different diameters. In this work, the author addresses these two difficulties.

There is evidently very little research reported in the literature pertaining to gloved hand impressions. To date most research has been focused on the visualization of latent fingerprints on different types of gloves, the detection of DNA traces on gloves, or aspects of the glove material (composition) itself [16–19]. The aims of the present study were to investigate whether additional information such as the stature of a suspect could be gleaned from glove prints, and whether obtaining glove prints from different objects of different diameters may add to their value.

## Materials and methods

The current study was conducted at the Forensic Medicine and Clinical Toxicology Department and Minia University Hospital, Faculty of Medicine, Minia University, Egypt, from February 2017 to August 2017. Patients’ relatives and visitors to outpatient clinics participated in the study. Each participant was asked to wear a latex glove on their right hand. A suitable glove size was selected based on the participant’s usual clothing size (small, medium, or large). Only right-handed individuals older than 21 years of age participated in the study. Other inclusion criteria were the absence of a history of hand trauma, surgery, and metabolic or bone diseases. The study included 160 participants, 80 of each sex.

### Acquiring glove impressions

Four objects of different diameters were prepared, a small wooden rod 2 cm in diameter, and three glasses with diameters of 4, 6 and 8 cm. Each object was wrapped in white paper before it was grasped by the volunteer. After applying ink the participants were asked to grasp each object. They were instructed to avoid forcibly gripping the objects. In the cases of the wooden rod and the 4-cm diameter glass an impression of the gloved thumb could not be obtained; therefore, thumb impression data for these objects were not included in the study. The participants were asked to keep their hands and forearms perpendicular to the grasped object. To achieve that positioning, the objects were placed on a table with a height that was suitable for each participant. In addition, glove prints of flat hands were obtained on white A4 paper.

In cases where a volunteer had evidently worn a larger than suitable glove as indicated by a hazy impression, the procedure was repeated after the volunteer donned a smaller glove. In cases where a part of the finger impression failed to appear, the subject was asked to repeat the process until fully visible glove impression marks appeared. In most cases prints were evident on the second trial.

### Stature estimation

Because there are diurnal variations in stature all measures were taken between 9 am and 11 am [20]. Each participant was asked to stand upright and barefoot on flat ground. An anthropometric measuring rod was placed straight up facing the participant. The participant was instructed to keep their feet parallel or slightly divergent, and their head maintained in the Frankfurt position [21]. Stature was measured twice and the mean was recorded to the nearest mm.

### Glove impression measurement

**Figure 1.**
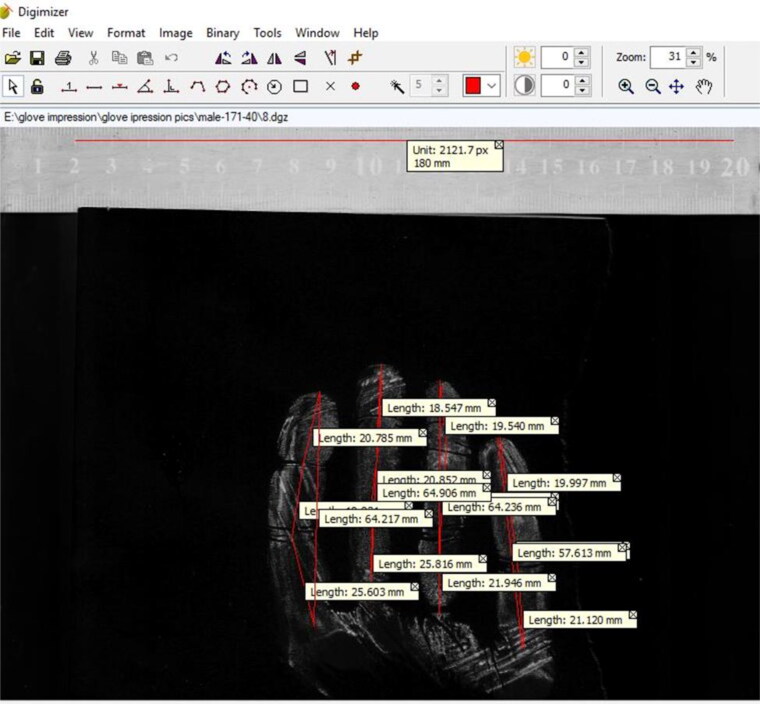
Digimizer program interface with the results of measurements of different glove impression dimensions.

After five glove prints were obtained from each participant the paper was removed from each object and scanned at 300 dpi (HP Scanjet 200 scanner). The resulting images were analysed via Digimizer 4.6.1 (2005–2017 MedCalc Software bvba, Ostend, Belgium), which is a free, easy-to-use and flexible image analysis software package that facilitates precise manual measurements and image manipulation. The images were converted to grayscale then inverted for additional enhancement. Calibration was performed using a scale inserted during image scanning. The following measurements were taken (Figure 1):

Finger length; where the length of each finger was measured as the distance from the furthest projecting point to the middle of the proximal phalangeal crease.

Phalangeal length; measured as the distance between the centres of two adjacent phalangeal creases. The distal phalange length was measured as the distance between the most forward-projecting points of the tip of a finger to the distal phalange crease.

### Statistical analysis

Means and standard deviations were obtained for all measures, including age and stature. Independent *t*-tests were conducted to compare mean stature and glove impression measures derived from female and male participants. Pearson’s correlation coefficient was used to assess relationships between stature and the measures obtained.

Stepwise discriminant function analysis was performed to investigate the capacity to predict sex from glove impressions. The leave-one-out cross-validation method was used to determine the accuracies of the derived discriminant functions. Stepwise multiple regression analysis was used to estimate stature using the measurements obtained. A generalized equation designed to predict the sex and stature of an offender from gloved handprints left while grasping objects with different diameters was developed. This was done by including object diameter as a variable. Stepwise regression equations were developed as follows: stature = constant + b1v1 + b2v2 + …, where b1 and b2 (*etc*.) are unstandardised coefficients and v1 and v2 (*etc*.) are the measures estimating stature. The standard error of the estimate (SEE) was used to measure the deviation of expected stature from actual stature. *F* value ≥ 3.84 indicated that the model was acceptable, and *F* ≤ 2.71 was grounds for rejection in both regression and discriminant analysis. These range standards were used by default by the SPSS program [22]. *P* < 0.05 was deemed to indicate statistical significance [23]. Statistical analysis was performed using SPSS v.23 (IBM, Armonk, NY, USA) for Windows on a personal computer.

## Results

**Figure 2.**
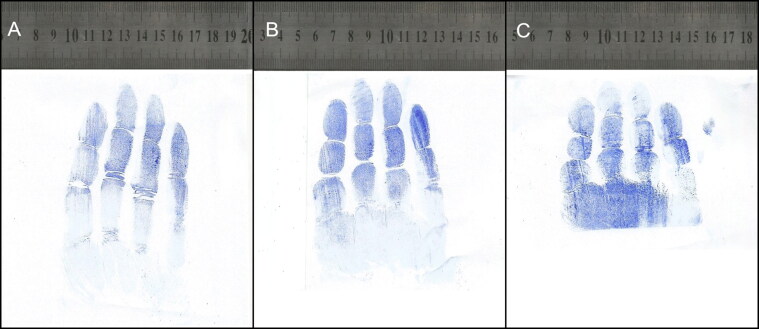
Glove impression marks when the same gloved hand grasped objects of 6 cm (A), 4 cm (B) and 2 cm (C) in diameter.

**Figure 3.**
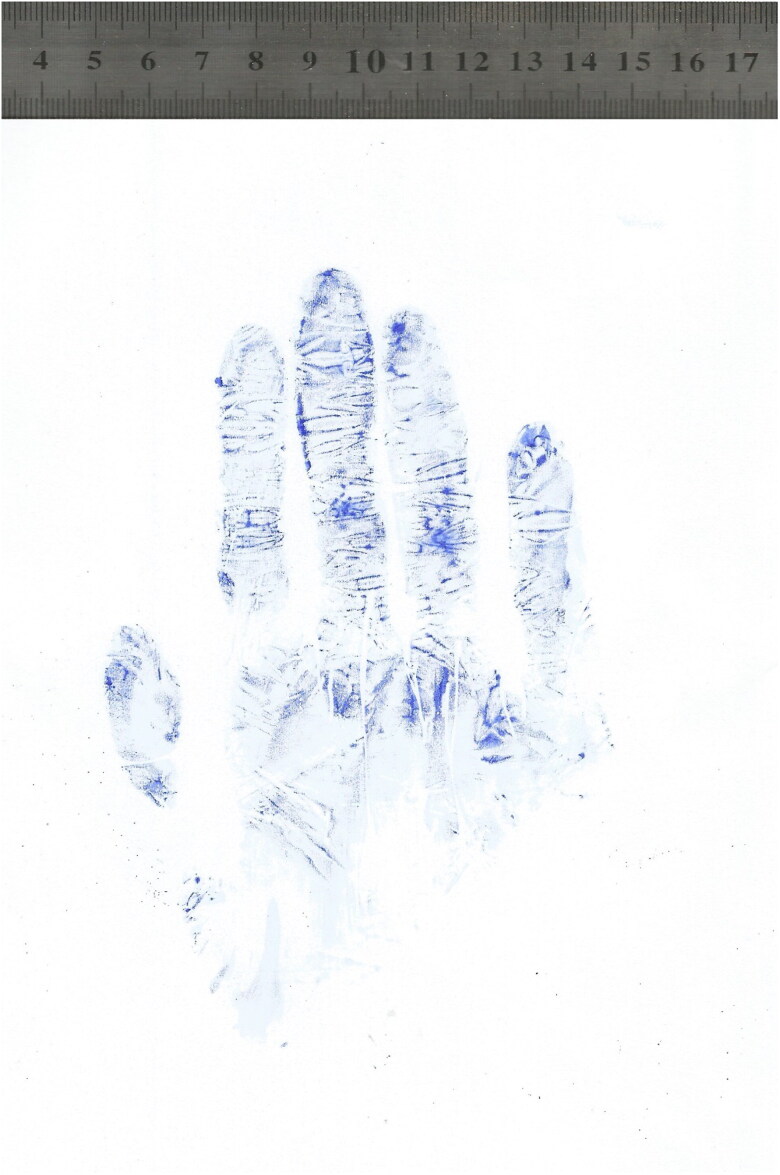
A glove impression derived from a participant who wore a glove that was larger than was suitable for him.

**Figure 4.**
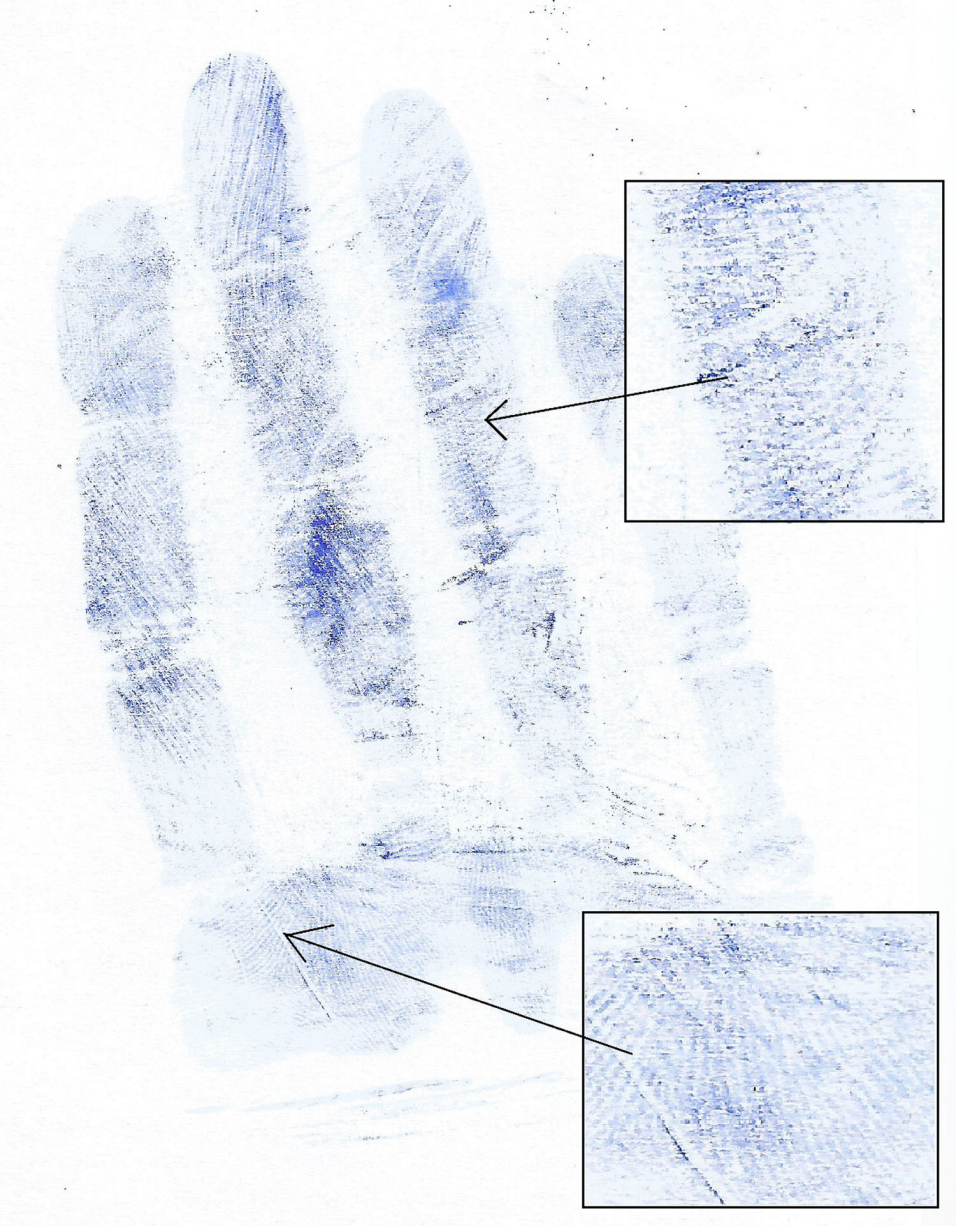
A glove impression mark showing the partial ridge prints of the participant.

**Figure 5.**
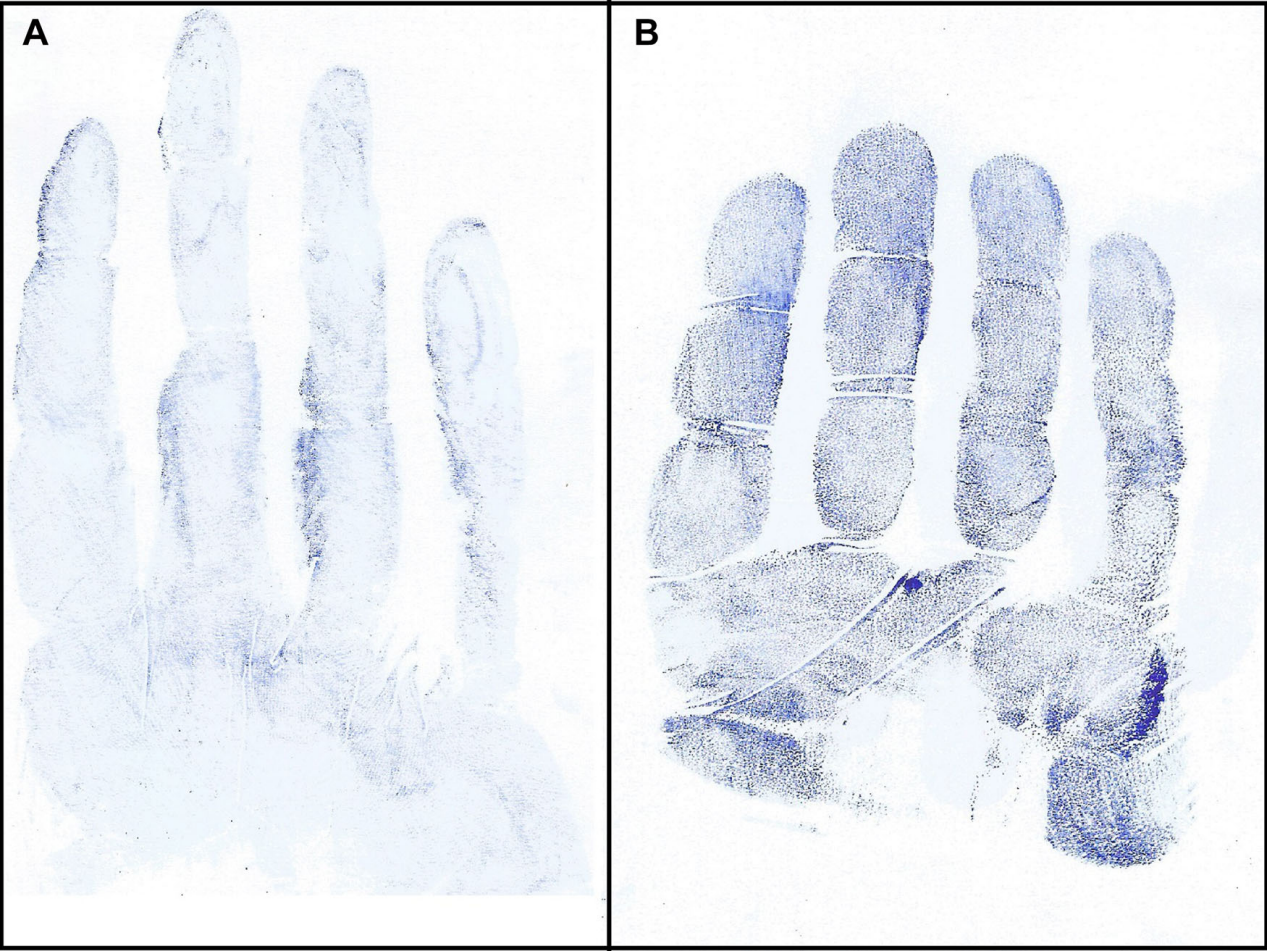
Impression marks from a gloved flat hand (A) and a gloved hand grasping an object 8 cm in diameter (B), in which well-demarcated phalangeal creases are evident in the right impression mark.

Impression marks left by gloved hands grasping objects of different diameters are shown in Figure 2. When a participant wore a glove that was too large the glove marks were distorted with corrugations, and it was difficult to identify the phalangeal crease (Figure 3). Sometimes partial ridge prints appeared in the glove impressions (Figure 4). The proximal phalange of the little finger was most frequently absent from a glove mark, requiring the subject to repeat the test. Phalangeal creases were clearer in the glove marks of grasped objects than those from flat hands (Figure 5).

With regard to the age distribution among the participants, the respective means and standard deviations for males and females were (40.8 ± 16.2) and (39.8 ± 13.5) years. The overall mean for both sexes was 40.3 ± 14.9 years (range 22–70 years). There was no significant difference in age distribution between males and females.

Handprint measurements of flat hands yielded measures that were significantly longer than the average. These results were true in cases when the volunteers were taller than the average. In addition, in the present study the mean height of males ((170.40 ± 6.70) cm) was significantly greater than that of females ((160.68 ± 6.90) cm). Therefore, longer handprints measurements of flat hands than the average suggested that the participants were mostly males. Furthermore, shorter handprints measurements of flat hands than the average suggested that the participants were mostly females. There were some exceptions to these general trends, resulting in some comparisons yielding insignificant differences between the two sexes or longer measurements in females than in males (Tables 1 and 2).

**Table 1.** Descriptive statistics derived from measurements of glove impressions made by flat hands. All measurements are in centimetres.

Fingers	Males		Females		*t*-values	*P* ^a^
	Mean ± SD	SEM	Mean ± SD	SEM		
L1	2.36 ± 0.29	0.02	2.12 ± 0.25	0.02	5.55	0.000
L2	1.75 ± 0.13	0.01	1.54 ± 0.22	0.02	7.77	0.000
L3	1.91 ± 0.44	0.03	2.02 ± 0.31	0.02	−1.77	0.078
L	6.09 ± 0.46	0.03	5.67 ± 0.42	0.03	6.15	0.000
R1	2.56 ± 0.20	0.01	2.39 ± 0.15	0.01	6.26	0.000
R2	2.39 ± 0.15	0.01	2.19 ± 0.24	0.02	6.48	0.000
R3	2.35 ± 0.40	0.03	2.57 ± 0.42	0.03	−3.29	0.001
R	7.35 ± 0.47	0.03	7.14 ± 0.55	0.04	2.72	0.007
M1	2.56 ± 0.15	0.01	2.38 ± 0.31	0.02	4.74	0.000
M2	2.54 ± 0.12	0.01	2.34 ± 0.31	0.02	5.48	0.000
M3	2.73 ± 0.34	0.02	2.86 ± 0.34	0.02	−2.39	0.018
M	7.93 ± 0.40	0.03	7.58 ± 0.56	0.04	4.60	0.000
I1	2.42 ± 0.14	0.01	2.22 ± 0.22	0.01	6.90	0.000
I2	2.38 ± 0.27	0.02	2.18 ± 0.22	0.02	5.16	0.000
I3	2.46 ± 0.28	0.02	2.45 ± 0.27	0.02	0.21	0.834
I	7.23 ± 0.36	0.02	6.84 ± 0.45	0.03	6.20	0.000

SD: standard deviation; SEM: standard error of the mean; L1, L2, and L3: distal, middle, and proximal phalanges of the little finger; R1, R2, and R3: distal, middle, and proximal phalanges of the ring finger; M1, M2, and M3: distal, middle, and proximal phalanges of the middle finger; I1, I2, and I3: distal, middle, and proximal phalanges of the index finger; L, R, M, and I: little, ring, middle, and index fingers.

^a^Significant at *P* < 0.05.

**Table 2.** Descriptive statistics for measurements of glove impressions from grasped objects of different diameters (means ± standard deviation). All measurements are in centimetres.

Fingers	Object’s diameter							
	8 cm		6 cm		4 cm		2 cm	
	Males	Females	Males	Females	Males	Females	Males	Females
L1	2.12 ± 0.15	1.91 ± 0.15	2.00 ± 0.24	1.68 ± 0.41	1.92 ± 0.25	1.59 ± 0.41	1.55 ± 0.15	1.46 ± 0.12
L2	1.56 ± 0.33	1.45 ± 0.18	1.42 ± 0.29	1.26 ± 0.42	1.28 ± 0.18	1.19 ± 0.30	1.04 ± 0.27^a^	0.99 ± 0.21
L3	1.46 ± 0.35	1.64 ± 0.43	1.39 ± 0.32^a^	1.32 ± 0.38	0.88 ± 0.42	1.31 ± 0.31	0.92 ± 0.31^a^	0.86 ± 0.29
L	5.18 ± 0.47	5.00 ± 0.39	4.68 ± 0.63	4.28 ± 1.08	4.32 ± 0.36	3.99 ± 0.96	3.52 ± 0.31	3.33 ± 0.36
R1	2.21 ± 0.21	2.05 ± 0.15	2.18 ± 0.21	1.95 ± 0.25	1.91 ± 0.16	1.85 ± 0.19	1.49 ± 0.20	1.41 ± 0.23
R2	2.15 ± 0.17	1.95 ± 0.16	1.82 ± 0.26	1.69 ± 0.18	1.67 ± 0.23	1.48 ± 0.27	1.33 ± 0.33^a^	1.26 ± 0.21
R3	1.85 ± 0.32	2.08 ± 0.15	1.55 ± 0.33^a^	1.62 ± 0.30	1.28 ± 0.35	1.51 ± 0.32	0.93 ± 0.34	1.06 ± 0.33
R	6.11 ± 0.34^a^	6.07 ± 0.33	5.32 ± 0.44^a^	5.25 ± 0.46	4.78 ± 0.35^a^	4.80 ± 0.44	3.69 ± 0.34^a^	3.72 ± 0.39
M1	2.16 ± 0.19	2.00 ± 0.18	2.04 ± 0.21	1.92 ± 0.30	1.80 ± 0.21^a^	1.81 ± 0.26	1.44 ± 0.15^a^	1.39 ± 0.27
M2	2.17 ± 0.17	2.02 ± 0.24	1.77 ± 0.34	1.68 ± 0.24	1.60 ± 0.27	1.46 ± 0.27	1.22 ± 0.29^a^	1.18 ± 0.19
M3	2.19 ± 0.32^a^	2.26 ± 0.25	1.68 ± 0.42^a^	1.78 ± 0.37	1.49 ± 0.29	1.68 ± 0.24	1.06 ± 0.36^a^	1.07 ± 0.32
M	6.46 ± 0.47	6.26 ± 0.35	5.11 ± 0.54	5.36 ± 0.51	4.82 ± 0.33^a^	4.87 ± 0.38	3.66 ± 0.42^a^	3.65 ± 0.39
I1	2.00 ± 0.15	1.90 ± 0.13	1.88 ± 0.22	1.80 ± 0.16	1.74 ± 0.24^a^	1.70 ± 0.20	1.28 ± 0.15^a^	1.32 ± 0.36
I2	1.90 ± 0.17	1.64 ± 0.12	1.56 ± 0.27	1.40 ± 0.23	1.39 ± 0.17	1.16 ± 0.19	1.10 ± 0.30	0.85 ± 0.30
I3	2.27 ± 0.35^a^	2.34 ± 0.27	1.84 ± 0.45^a^	1.93 ± 0.35	1.42 ± 0.36	1.63 ± 0.32	1.31 ± 0.37^a^	1.29 ± 0.38
I	6.02 ± 0.39	5.82 ± 0.37	4.81 ± 0.74^a^	4.89 ± 0.58	4.29 ± 0.63^a^	4.16 ± 0.99	3.65 ± 0.59	3.28 ± 0.90

L1, L2, and L3: distal, middle, and proximal phalanges of the little finger; R1, R2, and R3: distal, middle, and proximal phalanges of the ring finger; M1, M2, and M3: distal, middle, and proximal phalanges of the middle finger; I1, I2, and I3: distal, middle, and proximal phalanges of the index finger; L, R, M, and I: little, ring, middle, and index fingers.

^a^Insignificant difference (*P* ≥ 0.05) in comparison with corresponding measurements derived from females.

In comparisons of individual glove impression measurements in different hand positions when grasping objects of different diameters, there were significant positive correlation coefficients. Smaller object diameters were associated with smaller glove print measures. The ring and middle fingers yielded the highest correlations, with respective *r* values of 0.901 and 0.895 (Table 3).

**Table 3.** Correlation coefficients between object diameter and glove impression measurements.

Correlations	L1	L2	L3	L	R1	R2	R3	R	M1	M2	M3	M	I1	I2	I3	I
Pearson correlation	0.504^a^	0.552^a^	0.599^a^	0.629^a^	0.718^a^	0.743^a^	0.719^a^	0.901^a^	0.707^a^	0.778^a^	0.757^a^	0.895^a^	0.693^a^	0.727^a^	0.716^a^	0.768^a^
Sig. (two-tailed)	0.000	0.000	0.000	0.000	0.000	0.000	0.000	0.000	0.000	0.000	0.000	0.000	0.000	0.000	0.000	0.000

L1, L2, and L3: distal, middle, and proximal phalanges of the little finger; R1, R2, and R3: distal, middle, and proximal phalanges of the ring finger; M1, M2, and M3: distal, middle, and proximal phalanges of the middle finger; I1, I2, and I3: distal, middle, and proximal phalanges of the index finger; L, R, M, and I: little, ring, middle, and index fingers.

^a^Significant at 0.01 (two-tailed).

Stepwise discriminant analysis of glove prints from flat hands successfully determined sex with an overall accuracy of 84.6%. In males the accuracy was 92.3%, while in females it was 76.9%. The accuracy of sex estimation was markedly improved when glove impressions from grasped objects of different diameters were used rather than impressions from flat hands. When a generalised equation developed from stepwise discriminant analysis was applied for any object, however, the accuracy of sex determination dropped to 83.7%. A generalised equation developed from simple discriminant analysis applied for any object diameter failed to predict sex (Table 4).

**Table 4.** Discriminant function equations and cross-validated classification accuracies for the prediction of sex based on glove impressions from different hand positions.

OD	Equation^a^	Wilk’s lambda	Canonical correlation	Cross-validated, correctly classified (%)		
				Males	Females	Overall
Flat	*y* = 3.27 L2 + 0.77 L + 1.91 R2- 1.09 R- 1.33 M3 + 1.15 M + 4.12 I1- 2.43 I3-15.077	0.337^b^	0.814	92.3	76.9	84.6
8 cm	*y* = 3.83 L1 + 4.46 L- 6.11 R1-17.41 R2-28.95 R3 + 18.16 R- 4.60 M1 + 2.12 M3 + 1.29 M + 15.15 I1 + 26.26 I2 + 17.33 I3-18.67 I-44.379	0.166^b^	0.966	96.2	92.4	94.3
6 cm	*y* = 2.24 L3-0.97 L + 1.37 R1-10.79 R2-9.57 R3 + 13.12 R + 9.65 M2 + 6.17 M3 -11.69 M-1.67 I1 + 1.96 I- 9.244	0.268^b^	0.855	92.3	92.3	92.3
4 cm	*y* = 1.58 L1 + 6.95 L2-0.60 L3-2.04 L + 2.30 R1 + 1.87 R2- 2.78 M2 + 2.2 L3 M + 4.09 I1 + 11.38 I2-2.00 I-29.091	0.279^b^	0.849	91.6	90.2	90.9
2 cm	*y* = 5.72 L2-6.42 L1 + 3.87 L3-12.25 L-1.48 R2 + 4.01 R + 2.77 M3 + 2.45 M + 13.91 I1 + 11.71 I2-7.40 I + 13.763	0.284^b^	0.846	95.3	94.3	94.8
Equations for all objects	*y* = -0.570 OD + 0.144 L1 + 0.105 L2-0.375 R3 + 0.161 R + 0.128 M1 + 0.056 M2 + 0.166 M3-0.124 M + 0.289 I2-7.253	0.569^b^	0.656	84.6	82.7	83.7
	*y* = -0.348 OD + 0.428 L1-5.887	0.830	0.412	65.4	70.9	86.1
	*y* = -0.528 OD + 0.414 I2-3.021	0.805	0.441	73.1	63.5	68.3
	*y* = -0.165 OD + 0.411 L2-4.665	0.983	0.123	53.8	51.0	52.4

L1, L2, and L3: distal, middle, and proximal phalanges of the little finger; R1, R2, and R3: distal, middle, and proximal phalanges of the ring finger; M1, M2, and M3: distal, middle, and proximal phalanges of the middle finger; I1, I2, and I3: distal, middle, and proximal phalanges of the index finger; L, R, M, and I: little, ring, middle, and index fingers; OD: object’s diameter in cm.

^a^significant at *P* < 0.001.

^b^The discrimination point is zero (i.e. discriminant scores > 0 indicate males and scores < 0 indicate females).

Correlation coefficients between stature and glove impression measurements in various hand positions are shown in Table 5. Most of the measures were significantly correlated with stature, and some single measures (such as L1 and L2) yielded different correlations in different hand positions.

**Table 5.** Pearson’s correlation coefficients between measurements and stature in males and females (*r* values).

OD	Sex	L1	L2	L3	L	R1	R2	R3	R	M1	M2	M3	M	I1	I2	I3	I
Flat	Males	0.109^a^	0.257	0.158	−0.156	0.404	0.470	0.050^a^	−0.154	0.014^a^	0.471	0.114^a^	−0.004^a^	0.123	0.160	0.351	0.364
	Females	0.269	−0.224	0.068^a^	0.198	0.364	−0.302	0.522	0.381	0.326	0.159	0.464	0.608	0.496	0.269^a^	0.224^a^	0.068
8 cm	Males	0.311	0.328	−0.220	−0.127	0.151	0.118	−0.216	0.285	0.317	0.238	−0.043^a^	0.000^a^	0.506	0.587	0.449	0.231
	Females	0.203	0.056^a^	0.537	0.586	0.041^a^	0.141	0.435	0.298	0.171	0.244	0.165	0.403	0.133	0.186	0.091^a^	0.357
6 cm	Males	0.117	−0.195	0.408	0.065^a^	−0.217	−0.396	0.266	0.067^a^	0.050	0.456	0.325	−0.010^a^	0.342	0.357	0.041^a^	0.255
	Females	0.484	0.273	0.295	0.389	0.056^a^	0.061^a^	0.253	0.298	0.092^a^	0.134	0.240	0.300	0.103^a^	0.486	0.019^a^	0.396
4 cm	Males	0.166	−0.366	0.360	−0.210	0.019^a^	−0.334	0.487	0.275	0.005^a^	0.028^a^	0.237	0.256	0.311	0.378	0.459	0.619
	Females	0.338	0.603	0.046^a^	0.569	−0.203	0.253	0.197	0.390	0.045^a^	0.129	0.018^a^	0.184	−0.247	0.504	0.364	0.595
2 cm	Males	0.082^a^	0.182	0.098^a^	0.388	0.094^a^	0.092^a^	0.377	0.488	0.285	0.159	0.433	0.799	0.346	0.566	0.476	0.791
	Females	0.162	0.112^a^	0.356	0.176	0.009^a^	0.186	−0.249	0.044^a^	0.155	0.070^a^	0.508	−0.165	0.558	0.066^a^	0.519	0.303

L1, L2, and L3: distal, middle, and proximal phalanges of the little finger; R1, R2, and R3: distal, middle, and proximal phalanges of the ring finger; M1, M2, and M3: distal, middle, and proximal phalanges of the middle finger; I1, I2, and I3: distal, middle, and proximal phalanges of the index finger; L, R, M, and I: little, ring, middle, and index fingers; OD: object’s diameter in cm.

^a^Insignificant correlation coefficient (*P* > 0.05).

Simple linear regression equations for the estimation of stature from measurements of glove impressions from flat hands are shown in Table 6. Examples of simple linear regression equations for stature estimation based on measurements of gloved hand marks obtained from objects with diameters of 2, 4, 6 and 8 cm are shown in Table 7. Generalised equations developed from simple regression analysis in a trial investigating stature estimation based on measurements of gloved hand marks obtained from objects with any diameter are shown in Table 8. The SEE, which indicates the deviation of predicted values from actual measures, was slightly increased in the generalised equations. It ranged from 6.24 cm using the index finger in males to 6.96 cm using the ring finger in males. Only measurements that exhibited significant correlations with stature were calculated. To improve the ability to predict stature from glove impression measures, stepwise regression analysis was applied. Stepwise regression equations for stature estimation based on prints from flat gloved hands are shown in Table 9. The results of stepwise regression analysis of data derived from objects of the diameters investigated are shown in Table 10. Generalised stepwise regression equations for stature estimation based on measurements of impressions left by gloved hands grasping objects with any diameter are shown in Table 11. While stepwise analysis markedly improved stature prediction in cases where the individual diameters investigated were factored into the equations, generalised equations for any object diameter did not exhibit useful predictive value.

**Table 6.** Simple linear regression equations for stature estimation (in cm) from measurements of glove impressions from flat hands.

Sex	Equation	±SEE	*R* ^2^
Males	*y* = 194.482 + 13.10 L2	6.78	0.066
	*y* = 176.431 + 2.59 L3	6.92	0.025
	*y* = 186.143 – 2.41 L	6.92	0.024
	*y* = 207.958 + 14.31 R1	6.42	0.163
	*y* = 116.329 + 23.04 R2	6.19	0.221
	*y* = 188.749 – 2.36 R	6.93	0.024
	*y* = 98.698 + 28.67 M2	6.19	0.222
	*y* = 156.541 + 6.11 I1	6.96	0.015
	*y* = 160.912 + 4.44 I2	6.92	0.025
	*y* = 149.856 + 8.67 I3	6.57	0.123
	*y* = 118.632 + 7.26 I	6.53	0.133
Females	*y* = 144.019 + 7.48 L1	6.67	0.072
	*y* = 141.256 + 3.31 L	6.79	0.039
	*y* = 123.219 + 15.53 R1	6.45	0.133
	*y* = 179.137 – 8.86 R2	6.60	0.091
	*y* = 137.772 + 8.70 R3	5.59	0.273
	*y* = 126.391 + 4.73 R	6.40	0.145
	*y* = 142.552 + 7.37 M1	6.54	0.106
	*y* = 132.505 + 9.65 M3	6.13	0.215
	*y* = 108.003 + 6.93 M	5.49	0.370
	*y* = 124.973 + 15.89 I1	6.01	0.246
	*y* = 119.308 + 5.99 I	6.34	0.161

L1, L2, and L3: distal, middle, and proximal phalanges of the little finger; R1, R2, and R3: distal, middle, and proximal phalanges of the ring finger; M1, M2, and M3: distal, middle, and proximal phalanges of the middle finger; I1, I2, and I3: distal, middle, and proximal phalanges of the index finger; L, R, M, and I: little, ring, middle, and index fingers; SEE: standard error of the estimate (cm).

**Table 7.** Simple linear regression equations for stature estimation (in cm) from measurements of glove impressions of hand-gripped objects with each of the individually investigated diameters.

Sex	Object’s diameter	Equation	±SEE	*R* ^2^
Males	8 cm	*y* = 127.009 + 22.19 I1	6.05	0.256
		*y* = 216.329 + 23.79 I2	5.68	0.345
		*y* = 149.803 + 9.41 I3	6.27	0.202
	6 cm	*y* = 164.651 + 3.43 L1	6.96	0.014
		*y* = 184.525 + 9.54 L3	6.40	0.167
		*y* = 185.993 + 9.36 I2	6.55	0.128
	4 cm	*y* = 152.786 + 13.40 I2	6.49	0.143
		*y* = 158.022 + 1.014 R3	6.12	0.238
		*y* = 141.766 + 6.77 I	5.51	0.383
	2 cm	*y* = 134.414 + 99.9 R	6.12	0.238
		*y* = 121.265 + 13.62 M	4.22	0.638
		*y* = 136.079 + 9.60 I	4.29	0.625
Females	8 cm	*y* = 148.074 + 7.55 L3	5.84	0.288
		*y* = 111.096 + 9.92 L	5.61	0.343
		*y* = 115.506 + 21.51 R3	6.23	0.189
	6 cm	*y* = 148.842 + 6.69 L1	6.06	0.235
		*y* = 153.884 + 4.71 L3	6.62	0.087
		*y* = 140.604 + 14.14 I2	6.05	0.232
	4 cm	*y* = 146.397 + 11.61 L2	5.52	0.364
		*y* = 146.829 + 3.36 L	5.69	0.324
		*y* = 145.303 + 3.59 I	5.57	0.353
	2 cm	*y* = 170.580 + 10.1 M3	5.96	0.258
		*y* = 148.134 + 9.37 I1	5.74	0.311
		*y* = 171.165 + 9.03 I3	5.92	0.269

L1, L2, and L3: distal, middle, and proximal phalanges of the little finger; R1, R2, and R3: distal, middle, and proximal phalanges of the ring finger; M1, M2, and M3: distal, middle, and proximal phalanges of the middle finger; I1, I2, and I3: distal, middle, and proximal phalanges of the index finger; L, R, M, and I: little, ring, middle, and index fingers; SEE: standard error of the estimate (cm).

**Table 8.** Generalised simple linear regression equations for the estimation of stature (in cm) from measurements of glove impressions left by hands grasping any object.

Sex	Equation	±SEE	*R* ^2^
Males	*y* = 180.37 + 0.41 OD – 0.08 L2	6.70	0.084
	*y* = 175.87 + 0.39 OD – 0.33 R1	6.96	0.011
	*y* = 176.38 + 0.59 OD – 0.46 R2	6.93	0.029
	*y* = 164.08 + 0.93 OD – 0.24 R	6.96	0.017
	*y* = 165.73 – 0.55 OD + 0.45 M1	6.94	0.018
	*y* = 163.08 – 1.27 OD + 0.29 M	6.85	0.043
	*y* = 159.79 – 1.14 OD + 0.98 I1	6.63	0.103
	*y* = 166.54 – 0.87 OD + 0.53 I3	6.68	0.091
	*y* = 156.85 – 1.93 OD + 0.51 I	6.24	0.206
Females	*y* = 153.97 – 0.32 OD + 0.44 L1	6.82	0.026
	*y* = 155.29 – 0.11 OD + 0.39 L2	6.85	0.017
	*y* = 151.50 – 0.72 OD + 0.29 L	6.57	0.096
	*y* = 158.49 – 0.62 OD + 0.53 R2	6.81	0.028
	*y* = 149.14 – 1.33 OD + 0.35 R	6.73	0.051
	*y* = 152.73 – 1.03 OD + 0.23 M	6.82	0.026
	*y* = 154.94 – 0.44 OD + 0.43 I1	6.81	0.028
	*y* = 155.03 – 1.06 OD + 0.81 I2	6.64	0.077
	*y* = 152.41 – 1.43 OD + 0.33 I	6.34	0.161

L1, L2, and L3: distal, middle, and proximal phalanges of the little finger; R1, R2, and R3: distal, middle, and proximal phalanges of the ring finger; M1, M2, and M3: distal, middle, and proximal phalanges of the middle finger; I1, I2, and I3: distal, middle, and proximal phalanges of the index finger; L, R, M, and I: little, ring, middle, and index fingers; OD: object’s diameter in cm; SEE: standard error of the estimate (cm).

**Table 9.** Stepwise regression equations for the estimation of stature (in cm) from gloved impressions of a flat hand.

Sex	Equation	±SEE	*R* ^2^
Males	*y* = 0.813–44.418 M2 + 24.65 I1	5.41	0.402
	*y* = 11.587–54.10 M2 + 37.26 I1-7.43 L	4.52	0.585
	*y* = 25.35-71.13 M2-34.16 I1-14.26 L +10.24 L3	4.00	0.679
	*y* = 12.937-88.54 M2 + 31.47 I2-14.27 L + 19.13 L3-9.13 R	3.06	0.813
	*y* = 21.971 + 93.17 M2 + 31.77 I1-9.48 L + 18.4 L3-10.28 R-25.64 L2-9.8 LI	2.19	0.905
Females	*y* = 128.156 + 9.10 M-16.71 R2	4.06	0.658
	*y* = 111.018 + 9.17 M -16.46 R2 + 7.61 L1	3.60	0.732
	*y* = 133.034 + 12.75 M-21.37 R2 + 12.91 L1-20.91 R1	2.73	0.847
	*y* = 138.211 + 15.41 M-24.13 R2 + 16.28 L1- 23.40 R1- 9.25 I1	2.37	0.885
	*y* = 143.005 + 16.55 M-23.26 R2 + 16.44 L1- 24.60 R1-12.08 I1- 3.23 L3	2.28	0.894

L1, L2, and L3: distal, middle, and proximal phalanges of the little finger; R1, R2, and R3: distal, middle, and proximal phalanges of the ring finger; M1, M2, and M3: distal, middle, and proximal phalanges of the middle finger; I1, I2, and I3: distal, middle, and proximal phalanges of the index finger; L, R, M, and I: little, ring, middle, and index fingers; SEE: standard error of the estimate (cm).

**Table 10.** Stepwise regression equations for stature estimation (in cm) from measurements of glove impressions from hand-gripped objects with each of the fixed diameters investigated (8, 6, 4 or 2 cm).

Sex	Object’s diameter	Equation	±SEE	*R* ^2^
Males	8 cm	*y* = 184.841 – 13.68 I2 + 11.90 I3–9.65 L2	4.46	0.599
		*y* = 220.096 – 13.99 I2 + 14.82 I3– 12.12 L2 – 17.96 R2	3.51	0.753
	6 cm	*y* = 147.708 – 15.21 M2 + 16.21 I1 + 9.67 L1	4.65	0.472
		*y* = 155.717 – 14.14 M2 + 14.22 I1 + 12.13 L1 – 7.99 L3	4.07	0.667
	4 cm	*y* = 143.069 + 6.05 I – 21.70 L2 + 6.07 R	4.30	0.627
		*y* = 96.929 + 14.17 I – 34.24 L2 + 18.60 R – 23.02 I3	2.91	0.830
	2 cm	*y* = 114.067 + 19.36 M – 13.74 L2	3.05	0.809
		*y* = 113.273 + 22.86 M– 17.98 L2 – 8.28 L3	2.13	0.908
Females	8 cm	*y* = 99.571 + 2.13 L+ 14.08 L1 + 7.29 L3 + 7.93 L2	5.30	0.422
		*y* = 72.293 + 6.58 L3 + 12.43 L2 + 70.43 R3 + 9.54 I + 44.67 R1 – 19.67 M3 – 15.71 I3 – 25.17 R	4.57	0.580
	6 cm	*y* = 125.473 + 17.95 I2 + 9.9 L15 – 5.33 L2	4.66	0.551
		*y* = 98.541 + 15.77 I2 + 12.36 L1– 9.32 L2 + 5.91 R	4.15	0.644
	4 cm	*y* = 133.595 + 20.64 L2 + 8.70 I3 – 7.74 L1	4.38	0.603
		*y* = 118.388 + 22.45 L2 + 11.07 I3 – 9.37 L1 + 6.62 M1	4.13	0.648
	2 cm	*y* = 127.617 + 3.19 I1 – 23.53 I3 + 16.11 M	4.17	0.739
		*y* = 153.604 + 4.19 I1– 23.93 I3 + 15.88 M – 17.72 L1	3.56	0.775

L1, L2, and L3: distal, middle, and proximal phalanges of the little finger; R1, R2, and R3: distal, middle, and proximal phalanges of the ring finger; M1, M2, and M3: distal, middle, and proximal phalanges of the middle finger; I1, I2, and I3: distal, middle, and proximal phalanges of the index finger; L, R, M, and I: little, ring, middle, and index fingers; SEE: standard error of the estimate (cm).

**Table 11.** Generalised stepwise regression equations for stature estimation (in cm) from measurements of glove impressions of hand-grasping objects of any diameter.

Sex	Equation	±SEE	*R* ^2^
Males	*y* = 161.12 – 2.11 OD + 0.53 I – 1.0 L2 + 0.53 I1	5.50	0.374
	*y* = 168.06 – 1.44 OD + 0.48 I – 1.01 L2 + 0.87 I1 – 0.68 R1	5.40	0.41
	*y* = 166.21 – 1.12 OD + 0.79 I – 0.73 L2 + 0.45 I1–1.06 R1 + 1.0 M1 – 0.38 M – 0.31 L3	5.10	0.467
Females	*y* = 150.66 – 1.13 OD + 0.38 I – 0.47 I3 + 0.21 L	6.02	0.244
	*y* = 146.95 – 1.38 OD + 0.41 I – 0.59 I3 + 0.20 L + 0.55 R2 – 0.41 I2	5.90	0.276
	*y* = 144.39 – 2.09 OD + 0.54 I – 0.81 I3 + 0.23 R2 – 0.58 I2 + 0.55 L3 – 0.62 I1 + 0.73 M – 0.82 M3	5.70	0.321

L1, L2, and L3: distal, middle, and proximal phalanges of the little finger; R1, R2, and R3: distal, middle, and proximal phalanges of the ring finger; M1, M2, and M3: distal, middle, and proximal phalanges of the middle finger; I1, I2, and I3: distal, middle, and proximal phalanges of the index finger; L, R, M, and I: little, ring, middle, and index fingers; OD: object’s diameter in cm; SEE: standard error of the estimate (cm).

## Discussion

There is nothing more disappointing to fingerprint experts than finding glove marks at a crime scene, because it usually means their subsequent tasks will require much more effort than the standard process of comparing fingerprints found at a crime scene with those of a suspect [9]. Numerous studies [24, 25] have emphasized the usefulness of footwear in the estimation of sex and stature, prompting the question as to whether evidence derived from gloved hands may be similarly informative. Measurements derived from impressions of gloved females’ hands are smaller than those of corresponding measurements derived from males. This was the case for all measures derived from glove impressions from flat hands in the present study. These findings are consistent with previously reported data derived from direct measurements of hands [26] and measurements of handprints [27]. Notably, glove impressions left by male hands are routinely larger than those left by female hands when they are derived from grasped objects of different diameters.

Sex was successfully predicted from glove impressions derived from various hand positions, with an expected classification accuracy above 84.6%. The accuracy of sex estimations based on glove impressions differed for different hand positions. It was least accurate when the hand was flat (84.6%), and the most accurate results were derived from impressions taken when the hand grasped a small wooden rod of 2 cm in diameter (94.8%). Generalised equations (developed from stepwise discriminant analysis) applied for objects of any diameter succeeded in sex classification of glove impressions of the hand. These findings are consistent with previously reported results derived from direct hand measurements and handprints made by bare hands [14, 28, 29].

Correlation coefficients between individual glove impression measurements and stature differed depending on hand position. A single measurement may be strongly and significantly correlated with stature in one particular hand position but weakly or insignificantly correlated with stature in another position. For example, L1 measurements were significantly positively correlated with stature when they were derived from females’ hands gripping an object 6 cm in diameter, but L1 measurements were not significantly correlated with stature when they were derived from flat hands of male subjects or males’ hands gripping a 2-cm diameter wooden rod. This highlights the fact that changes in hand position can have significant effects on glove impression measurements. These different correlations between stature and individual glove print measurements in different hand positions affect stepwise regression analysis equations. Different variables were used for various hand positions. Notably however, these variations do not affect the ability to predict stature from glove impressions. The SEEs associated with different hand positions were comparable. To a degree generalised equation developed via simple regression analysis were able to estimate stature based on objects with any diameter, but the associated SEE was slightly higher than that associated with equations developed using a specific object diameter. When more than one variable was used in regression equations the prediction of stature was more precise. This was true of all the glove impressions in all the different hand positions. These results are consistent with previously reported results derived from direct hand measurements and from handprint measurements [5, 13, 30, 31].

Some observations in the present study were not amenable to statistical analysis but warrant reporting because they are potentially broadly informative. One is that in cases where test participants wore gloves that were too large, glove impressions tended to exhibit wrinkles and corrugations and it was difficult to determine the sites of finger crease marks. Accordingly, forensics experts may consider the possibility that a criminal wore gloves that were too large if glove impressions from a crime scene exhibit wrinkles and corrugations and it is difficult to determine the sites of finger crease marks. Another potentially informative observation was the presence of partial finger ridge marks in some cases, and this has also been reported previously by Coppock [32].

In the current study finger creases were clearer in glove impressions derived from flexed fingers (as when a hand grips an object) than from impressions derived from flat hands. A possible explanation for this is that the glove partially obscured the finger creases when the hand was flat, but when the hand starts to flex, the creases become more pronounced. Impressions obtained from objects with smaller diameters yielded more predictive results than those obtained from flat hands or objects with larger diameters. This may be because finger crease marks become more evident when the hands are more flexed.

Glove impression measurements have yielded encouraging results with regard to predicting both sex and stature. Notably however, the need to change the equations, individual measures, and the constant used based on changes in hand position renders the use of glove impressions somewhat tedious. Another limitation associated with the forensic use of glove marks is that they are usually fragile and easily destroyed if not handled carefully [6].

## Conclusion

To the author’s knowledge, this is the first experimental study investigating gloved handprints in different positions. The results of the study provide insight into predictions of sex and stature based on numerous parameters of glove prints. This is a fundamental issue in crime scene investigations because criminals now frequently try to avoid being identified via their fingerprints by wearing gloves while committing crimes. Future studies investigating the effects of different types of gloves of various thicknesses would constitute valuable contributions to the collective knowledge-base.
